# Liquid–Liquid Phase Separation and Protective Protein Aggregates in Bacteria

**DOI:** 10.3390/molecules28186582

**Published:** 2023-09-12

**Authors:** Dorota Kuczyńska-Wiśnik, Karolina Stojowska-Swędrzyńska, Ewa Laskowska

**Affiliations:** Department of General and Medical Biochemistry, Faculty of Biology, University of Gdansk, Wita Stwosza 59, 80-308 Gdansk, Poland; dorota.kuczynska-wisnik@ug.edu.pl (D.K.-W.); karolina.stojowska-swedrzynska@ug.edu.pl (K.S.-S.)

**Keywords:** liquid–liquid phase separation, membraneless organelles, protein aggregates

## Abstract

Liquid–liquid phase separation (LLPS) and the formation of membraneless organelles (MLOs) contribute to the spatiotemporal organization of various physiological processes in the cell. These phenomena have been studied and characterized mainly in eukaryotic cells. However, increasing evidence indicates that LLPS-driven protein condensation may also occur in prokaryotes. Recent studies indicate that aggregates formed during proteotoxic stresses may also play the role of MLOs and increase the fitness of bacteria under stress. The beneficial effect of aggregates may result from the sequestration and protection of proteins against irreversible inactivation or degradation, activation of the protein quality control system and induction of dormancy. The most common stress that bacteria encounter in the natural environment is water loss. Therefore, in this review, we focus on protein aggregates formed in *E. coli* upon desiccation–rehydration stress. In silico analyses suggest that various mechanisms and interactions are responsible for their formation, including LLPS, disordered sequences and aggregation-prone regions. These data support findings that intrinsically disordered proteins and LLPS may contribute to desiccation tolerance not only in eukaryotic cells but also in bacteria. LLPS-driven aggregation may be a strategy used by pathogens to survive antibiotic treatment and desiccation stress in the hospital environment.

## 1. Introduction

**Figure 1:**
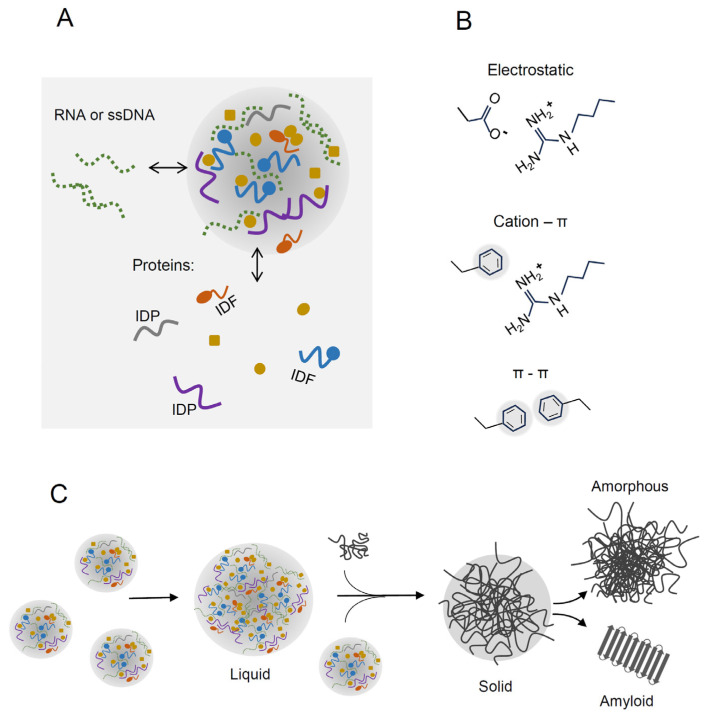
Liquid–liquid phase separation (LLPS) of macromolecules. (**A**) During LLPS, a homogenous solution of macromolecules is separated into dense liquid droplets and a diluted phase. Liquid droplets are dynamic and exchange their components with the diluted phase (IDP, intrinsically disordered protein; IDF, intrinsically disordered fragment). (**B**) Multivalent interactions in liquid droplets include electrostatic, cation–π and π–π interactions. (**C**) Smaller droplets may fuse into larger condensates. Upon prolonged stress, the liquid condensates may be irreversibly transformed into aberrant solid aggregates with amorphous and/or amyloid structures. During the transition from liquid to solid state, additional droplets, oligomers or small aggregates can be incorporated into larger aggregates.

Increasing evidence has shown that the liquid–liquid phase separation (LLPS) process is crucial to form multiple assemblies within the cell. During LLPS, a homogenous solution of macromolecules is separated into macromolecule-rich liquid droplets and a diluted phase (Figure 1A) [1,2]. Condensed liquid droplets form coacervates or membraneless organelles (MLOs). The best-characterized eukaryotic MLOs include the centrosome, the nucleolus, nuclear speckles, paraspeckles, Cajal bodies, processing bodies and stress granules which play a crucial role in a variety of biological processes [3,4,5,6]. Proteome-wide analysis revealed that spontaneous droplet separation may occur in the case of as much as 40% of the human proteome [7]. In general, condensates are formed in response to changing and stressful environments, and some of their main components are proteins with intrinsically disordered sequences and nucleic acids. These macromolecules usually play functions of scaffolds or LLPS-drivers essential for structural integrity of MLOs. Other components, including globular proteins, can also be recruited into MLOs as client proteins [1,2,3,8,9]. Several studies have reported that the formation of condensates via LLPS may also occur in bacteria despite the relatively low content of disordered sequences in microorganisms (18–28%) compared to multicellular eukaryotes (35–45%) [10,11,12,13]. Direct observations of LLPS or MLOs in vivo are difficult, primarily due to the small size of the bacteria. Hence, the prevalence of LLPS-driven condensates in bacteria may be underestimated. However, the list of bacterial proteins that undergo liquid separation is still being extended due to recent great progress in developing high-resolution microscopy and single-cell tracking techniques [11,14]. Recent studies suggest that LLPS and liquid droplets are the initial stages of protein aggregates that may protect bacteria and increase their fitness under proteotoxic stresses [15,16,17]. It is worth noting that the aggregates sequester hundreds of proteins [16,18,19], in contrast to most known bacterial MLOs, which usually contain several well-defined components [10,20].

This review will present background information on LLPS (Section 2), LLPS-prone bacterial proteins (Section 3) and bacterial protein aggregates formed upon proteotoxic stresses (Section 4). We will focus on aggregates formed in *E. coli* upon desiccation–rehydration stress, assuming that water loss and cytoplasm condensation under these conditions may facilitate LLPS and the formation of protective aggregates. Numerous reports have indicated that intrinsically disordered proteins (IDPs) and LLPS contribute to desiccation tolerance in eukaryotes [21,22,23,24,25] and bacteria [26,27]. Understanding mechanisms underlying bacterial desiccation tolerance is particularly important in the case of pathogens that can persist in an anhydrobiotic state in the hospital environment. LLPS and protective aggregates may also protect foodborne pathogens during the antimicrobial procedures used in the food processing industry, such as desiccation or freeze-drying [28,29]. Another interesting aspect is the link between protein aggregation, dormancy and antibiotic tolerance (Section 4.1).

## 2. Liquid–Liquid Phase Separation of Proteins

LLPS is driven by weak multivalent interactions, including electrostatic interactions, cation–π, π–π and other hydrophobic contacts, oligomerization domains, motif-binding domains, helix–helix interactions and β-zippers [30,31,32,33] (Figure 1B). Liquid droplets, coacervates or condensates are dynamic structures and macromolecules may diffuse between the phases. LLPS can be modulated by temperature, ionic strength, molecular crowding and post-translational modifications (PTMs) [34,35,36]. Crowding agents may stimulate separation, causing various effects, so the lower protein concentration is sufficient to form droplets. Volume exclusion by crowding agents enhances intermolecular interactions between macromolecules. A crowding agent may also directly interact and co-condensate with proteins or affect protein solubility.

### 2.1. Intrinsically Disordered Proteins

Structural disorder is a common feature of proteins capable of LLPS [32]. Although intrinsically disordered proteins (IDPs) and intrinsically disordered fragments (IDFs) are devoid of stable secondary or tertiary structures, they play essential biological functions, including signaling, regulation and recognition. IDP/IDF flexibility enables interactions with multiple ligands and facilitates post-translational modifications to a greater extent than defined three-dimensional protein structures [37,38]. Therefore, IDPs are often regarded as multifunctional proteins. Upon binding to ligands, IDPs undergo a disorder-to-order transition called induced folding [39,40,41]. Multiple studies have demonstrated that IDPs are involved in desiccation tolerance in plants and animals. IDPs may form shields around proteins and glass-like protective matrices or act as molecular chaperones [22,24,25,42].

The proteomes of different species comprise a whole spectrum of conformations from fully structured to completely disordered [38]. The analysis of entire proteomes of 3484 species from all domains of life and virus proteomes revealed that Archaea and bacteria have lower disorder content than eukaryotes, whereas the widest range of disorder (from ~7% to 77%) of disordered residues characterizes viruses [13]. Increased disorder in eukaryotic and some virus proteomes seems linked to increased cell complexity and a requirement for more complex signaling and regulations. The wide variation of disorders in unicellular eukaryotes may reflect the high variability of their habitats. Most bacteria possess a relatively small fraction of disordered residues, ranging from 18% to 28% [13]. This may explain why LLPS-driven condensates in bacteria are not as frequent as in eukaryotic cells.

### 2.2. Post-Translational Modification of Proteins in LLPS

There are multiple examples of condensates formed by LLPS and regulated by PTMs in eukaryotic cells [35,36,43]. Phosphorylation of proteins can inhibit or accelerate LLPS since the additional negative charge of the phosphoryl group may enhance electrostatic repulsion or attraction depending on the protein sequence. For instance, phosphorylation of three serine residues in a highly disordered C-terminal tail of histone H1 reduces its phase separation, affecting chromatin condensation [44]. Gibson et al. reported that chromatin undergoes LLPS in the presence of histone H1 in vitro and produces dynamic droplets after microinjection into cell nuclei. Acetylation of H1 inhibited LLPS and decreased droplet density. In mammalian cells, the protein arginine methylation occurs preferentially within the proteins identified in stress granules (SGs) and other MLOs. The global inhibition of arginine methylation promotes the formation of SGs and impaired self-disassembly of SGs [45].

PTMs of proteins in bacteria are less abundant and more diverse than in eukaryotic cells [46]. Low stoichiometry of PTMs is the main reason for difficulties in analyzing bacterial PTMs’ role. Nevertheless, over the last decade, numerous PTMs and their contribution to different physiological processes have been reported [46,47]. Several *E. coli* proteins that can form liquid droplets (see the next section) are post-translationally modified: SSB and TmaR are phosphorylated [47,48], and α, β and β’ subunits of RNAP are acetylated [49,50]. The exact contribution of these modifications in the formation of bacterial condensates has been reported only in the case of TmaR [48]. Since some PTMs change protein conformation or surface charge, they may affect interactions with other proteins or ligands. For example, the neutralization of lysine residues by acetylation increases the hydrophobicity of the lysine side chain, which in turn enhances the aggregation of endogenous proteins in *E. coli* [51]. However, it remains unknown whether LLPS is engaged in this process.

### 2.3. Transition of Liquid Droplets into Solid Aggregates

Depending on conditions, condensation may lead to the formation of highly ordered structures such as glassy solids, amyloid fibers or even crystals. In contrast to dynamic liquid droplets, these states are mostly irreversible (Figure 1C). Several studies have revealed that liquid droplets are the initial stage toward these stable forms in eukaryotes [1] and in bacteria [16]. Notably, the conversion of liquid droplets into a solid-like state can underline disease-associated amyloid formation. For example, FUS, a prion-like protein associated with amyotrophic lateral sclerosis, forms liquid compartments both in vivo and in vitro. In vitro, liquid droplets of FUS protein are converted with time to an aggregated state [52]. Ray et al. demonstrated that alpha-synuclein, a natively disordered protein associated with Parkinson’s disease, forms liquid droplets that are transformed into perinuclear aggresomes [53]. It was also found that soluble amyloid β oligomers undergo LLPS and are converted into amyloid fibrils—the hallmarks of Alzheimer’s disease [54]. LLPS of type II diabetes-associated IAPP protein promotes the formation of gel-like droplets, which are then irreversibly transformed into amyloid aggregates [55].

## 3. LLPS-Prone Proteins Participating in Physiological and Stress-Protecting Processes in Bacteria

Bacteria do not contain membrane-bound organelles, but some of them possess microcompartments with an enzymatic core encapsulated in a selectively permeable protein shell [56]. Polyhydroxybutyrate granules, storage compartments of carbon and energy in Eubacteria and Archaea, have been found to be covered with a layer of functional and structural proteins [57]. Apart from microcompartments, cardiolipin domains in the *E. coli* membrane also contribute to the differentiation of cell structure [58]. LLPS, as an alternative mechanism, may additionally influence the spatiotemporal and functional organization of the bacterial cell. LLPS participates in many processes occurring in bacteria: plasmid and chromosome partitioning (ParABS system), transcription (RNA polymerase clusters), cell division (FtsZ), DNA replication and repair (SSB), protection and adaptation to changing conditions (Dps, TmaR) [59,60,61,62,63]. Because comprehensive reviews on LLPS and MLOs in bacteria are available [10,11,20], we will present only proteins detected in *E. coli* aggregates formed upon desiccation–rehydration stress (see Section 4.1.1).

FtsZ assembly. FtsZ is a tubulin homolog that assembles into the Z ring at the site of cell division. Monterroso et al. reported that FtsZ from *E. coli* forms condensates in vitro in crowded cell-like conditions. The condensation occurs in the presence of SlmA (a DNA-binding protein and FtsZ polymerization antagonist) and SBS—the SlmA-binding sequence [61]. The presence of the SBS sequence in the condensates is in line with other findings indicating that nucleic acids may promote phase separation.

Single-stranded DNA binding protein (SSB). SSB is another bacterial protein that forms liquid–liquid phase separated coacervates [63]. Each subunit of the *E. coli* SSB homotetramer possesses an N-terminal DNA domain containing a single OB-fold, an intrinsically disordered linker (IDL) and a C-terminal protein–protein interaction peptide (CTP) [64]. SSB forms filaments and covers single-strand DNA to prevent a nucleolytic attack and aberrant intra-strand interactions during various DNA metabolic processes. Harami et al. showed that *E. coli* SSB forms viscous, liquid-state protein droplets in vitro under physiological concentrations of ions and protein [63]. All structural SSB regions participated in the phase separation; SSB tetramers are condensed via multivalent interactions between the IDL regions and interactions between the CTPs and OB folds. Since ssDNA binds to the OB-fold, it outcompetes the CTPs and inhibits phase separation. Based on these results and previous findings, the authors proposed that SSB and SSB-interacting clients, including DNA-repair proteins, are stored in the condensed form at the inner membrane. An increase in the level of ssDNA during stress leads to the dissolving of the SSB condensates. Thus, SSB and DNA-repair enzymes are mobilized to target DNA damage sites rapidly [63].

RNA polymerase clusters. Ladouceur et al. demonstrated that during the transition from lag phase to log phase, *E. coli* RNA polymerase (RNAP) formed condensates sensitive to hexanediol, which dissolves liquid-like compartments in eukaryotic cells [58]. RNAP clustering was mediated by protein–protein interactions rather than DNA binding, and required the antitermination factor NusA. Single-molecule tracking revealed that RNAP and NusA moved inside the clusters faster than a DNA locus but slower than molecules in the bulk nucleoid. All these results indicated that RNAP clustering occurred via LLPA.

HU and Dps. The most abundant nucleoid-binding proteins in *E. coli*, HU-A, HU-B and Dps, can form coacervates with different forms of DNA and RNA [12,62]. It was demonstrated that individually and collectively, HU-A, HU-B and Dps cause condensation of nucleic acids into globular phase-separated coacervates under conditions mimicking the cytosol of *E. coli* cells [62]. Dps and HU-B are overexpressed in response to starvation and are the main DNA-binding proteins in the stationary phase. During starvation, Dps forms tightly packed DNA co-crystals in *E. coli* cells. In addition, Dps is an iron-storage protein with ferroxidase activity. These Dps functions, ferrous ions scavenging and DNA compaction, protect bacteria against various stress factors [65].

The proteins described above have been detected as components of aggregates that contain hundreds of proteins isolated from *E. coli* exposed to proteotoxic stresses [18,19]. The next section will discuss whether such protein-abundant aggregates may also play a role of protective MLOs.

## 4. Protein Aggregation in Bacteria as a Consequence of Proteostasis Disruption

Proteostasis (i.e., maintaining the proper balance between protein synthesis, folding, localization and degradation) is crucial for optimal cell growth. Intrinsic or environmental stresses such as heat shock, desiccation, antibiotics, oxidative stress or metal ion exposure often disturb proteostasis, which is manifested by protein aggregation [19,66,67,68,69]. Genetic mutations and mistranslation may prevent the proper folding of polypeptides, leading to the exposure of hydrophobic amino acid residues. A similar effect may be caused by adverse environmental conditions that destabilize partly folded or native proteins. Nonnative interactions between folding intermediates and unfolded or misfolded proteins mediate aggregation. The exposed hydrophobic fragments, so-called aggregation-prone regions (APRs), promote the formation of intermolecular β structures [70]. Therefore, misfolded proteins in bacteria can form amyloid-like structures characterized by cross β-sheet interactions, similar to eukaryotic amyloids or amorphous aggregates. Examples of amyloid aggregates in bacterial cells are inclusion bodies (IBs) formed during high-level production of recombinant proteins [71,72]. It is worth noting that protein aggregation is sequence-specific and favors self-assembly rather than co-precipitation of mixed non-homologous sequences [73]. It has been demonstrated that APRs can be used as antibacterial agents [74,75]. Peptides containing a tandem of APRs were exploited to induce massive aggregation and formation of lethal inclusion bodies containing hundreds of bacterial proteins in *Staphylococcus aureus*, *E. coli* and *A. baumannii*. The peptides were effective against drug-resistant clinical isolates and reduced bacterial load in a murine infection model [74,75].

Bacteria have evolved a complex quality-control machinery, comprising molecular chaperones and proteases, to cope with the detrimental effects of stressful conditions and maintain proteostasis [76,77]. In *E. coli*, the main DnaK-DnaJ-GrpE chaperone system and the GroEL-GroES chaperonin are responsible for ATP-dependent refolding of denatured proteins, whereas proteases (ClpAP, ClpXP, HslUV, Lon) degrade irreversibly damaged proteins [69,77]. Under severe stress conditions, the quality control system is overloaded, leading to the accumulation of misfolded and aggregated proteins.

Bacterial populations can diminish toxic effects caused by misfolded proteins by asymmetrical segregation of aggregates during cell division. It has been demonstrated that protein aggregates formed during heat shock and in aging *E. coli* cells accumulated mainly at the cell poles [78,79]. According to Winkler et al., the polar localization of aggregates was caused solely by nucleoid occlusion [78]. However, other studies suggested that proton motive force, the DnaJ/DnaK chaperones, and an actin homolog MreB are required to transport aggregates to the poles [80]. It has been found that upon cell division, aggregates were segregated asymmetrically, appearing at the old pole, and those cells that inherited aggregates exhibited reduced growth rate [78,79]. This strategy, whereby dividing cells segregate damage at the expense of one subpopulation, helps the whole population persist under aging or environmental stresses.

Recent studies indicate that protein aggregates can have protective functions despite the detrimental effects of protein misfolding. This topic will be presented in the next section, with a particular focus on aggregates formed during desiccation–rehydration stress.

### 4.1. Protective Aggregates and LLPS

It is worth noting that aggregates formed upon proteotoxic stresses sequester hundreds of proteins [16,18,19], in contrast to most known bacterial MLOs, which usually contain one or few components (Section 3). Examples of bacterial protective aggregates are summarized in Table 1. Damaged proteins trapped in the aggregates do not interact with soluble macromolecules or membranes and are thus less toxic than misfolded but soluble oligomeric intermediates. It should also be noted that the aggregates, including IBs, may contain proteins with different conformations from totally unfolded to partially or even fully native structures. The presence of active enzymes in IBs has been reported in several studies [71,81,82,83]. Therefore, after the stress conditions cease, the aggregates can be used as a source of easily available functional proteins, which is a better strategy than de novo protein synthesis.

An interesting example of protective aggregates are “memory” inclusions formed in *E. coli* exposed to sublethal proteotoxic stresses such as high temperature, peroxide and streptomycin [15] (Table 1). After the removal of stress factors and the growth resumption, the aggregates located near the cell poles were only partly disaggregated. Similarly to earlier presented results [78,79], aggregates were asymmetrically inherited, giving rise to a heterogeneous population. Those cells that inherited the aggregates were able to cope better with the second stress exposure than their siblings devoid of aggregates. Thereby, the aggregates fulfilled the function of long-term epigenetic “memory” factors, which could persist even over several generations. The co-localization of molecular chaperones: DnaK, DnaJ, ClpB and the ClpP protease in the aggregate-bearing cells indicated that the mechanisms underlying increased stress tolerance involved the main elements of the protein quality control system in *E. coli* [15].

Aggregates of endogenous proteins were also detected in *Acinetobacter baumannii* exposed to desiccation stress [17]. It was found that the aggregates were associated with the ability of *A. baumannii* to survive desiccation. *A. baumannii* also acquired tolerance to the stress when protein aggregation was induced before desiccation by streptomycin treatment (a ribosome-targeting antibiotic that increases protein mistranslation and aggregation) or the *lon* gene deletion resulting in diminished degradation of misfolded proteins. Moreover, using β-galactosidase as a model enzyme, Wang et al. showed that proteins sequestered in the aggregates may retain their activities [17]. Therefore, it was proposed that the aggregates may contribute to desiccation tolerance in *A. baumannii* by preserving and protecting proteins. Apart from the direct effects of the aggregates, concomitant upregulation of several molecular chaperones in *A. baumannii* enabled the maintenance of proteostasis during desiccation, similar to aggresome-accumulating *E. coli* cells [16].

A correlation between the aggregation of endogenous *E. coli* proteins and the generation of persister bacteria is another example of protective mechanisms in bacteria exposed to antibiotics [85,86]. Persisters are non-growing, dormant cells that usually constitute a small fraction of the bacterial population and can survive high concentrations of antibiotics [87,88]. Since most antibiotics target processes that occur only in metabolically active cells (translation, replication or cell wall synthesis), dormant persisters are resistant to antibiotics. However, in contrast to resistant mutants, persisters are only phenotypic variants of wild-type bacteria and after resuming growth, they become drug-sensitive again. We have previously demonstrated that the frequency of persisters correlates with the level of protein aggregates formed during the stationary phase [84]. The formation of protein aggregates was modulated by osmolytes, MOPS buffer or sodium acetate without affecting the growth rate. When growth media were supplemented with low concentrations of osmolytes (trehalose, betaine, glycerol or glucose), proteins were prevented from aggregation and persister formation was inhibited. On the other hand, protein aggregation and persister formation were enhanced in the presence of acetate or high concentrations of osmolytes [84]. The elongation factor EF-Tu, one of the most abundant *E. coli* proteins, was particularly prone to aggregation during the stationary phase and was the main component of the aggregates. Thus, the inhibition of translation and other processes caused by the sequestration of EF-Tu and other essential proteins resulted in dormancy and the generation of persisters. It should be noted that protein aggregation is only one of the mechanisms linked to persister formation. Other factors and processes promoting the generation of persisters are described in several excellent reviews [85,87,88].

Further studies using a single-cell approach [18] confirmed the association of aggregates with persisters. Pu et al. demonstrated that the “dormancy depth” of *E. coli* cells increased in correlation with the fraction of insoluble proteins forming “aggresomes”. The aggresomes contained numerous *E. coli* proteins involved in a range of essential processes. It was proposed that the main cause of protein aggregation was ATP depletion. ATP level reduction diminished the efficiency of ATP-dependent molecular chaperones and proteases responsible for the removal of damaged proteins. The other possibility is that ATP also acts as a hydrotrope and prevents the formation of aggregates [89]. Pu et al. found that the resuscitation of persisters requires the removal of aggregates by ATP-dependent molecular chaperones DnaK and ClpB [18]. Further studies revealed that the aggresomes accumulated during a prolonged stationary phase not only in *E. coli* but also in other Gram-negative bacteria [16]. Inhibition of aggresome formation in respiration-impaired *E. coli* mutants, which sustained high ATP levels, led to an increased sensitivity to antibiotics and P1 phage infection. Similar effects, reduced aggresome formation and decreased bacterial fitness, were observed in *E. coli* cultures supplemented with MOPS. Most importantly, high-resolution optical microscopy revealed that aggresomes were formed through LLPS. As in other MLOs, aggresome components were mobile, underwent turnover, and initial liquid droplets were fused into larger dynamic condensates [16].

#### 4.1.1. Analysis of *E. coli* Protein Aggregates Formed during Desiccation–Rehydration Stress

Our previous studies revealed that protein aggregates formed in *E. coli* during desiccation–rehydration stress were enriched in proteins prone to liquid–liquid phase separation [19]. Proteins with a high tendency to undergo LLPS (catGranule propensity score greater than one) belonged to different groups, including ribosomal proteins, enzymes involved in the TCA cycle, fatty acid biosynthesis, and membrane assembly. We also compared the aggregated proteins and the entire *E. coli* proteome using the D^2^P^2^ platform, which detects disordered sequences [90]. Some disorder predictors (VXLT, VSL2B) indicated the overrepresentation of IDPs in aggregates, but other algorithms (PrDos and EspritzD) revealed less significant differences. We supposed that the aggregates were formed by a similar mechanism as previously described aggresomes [16]. Apart from protein aggregation, another effect of desiccation–rehydration stress was enhanced proteins’ non-enzymatic glycosylation (glycation). Although it is known that glycation may induce protein aggregation in vitro [91,92,93], we found that glycation did not stimulate the formation of *E. coli* aggregates. Glycation products were detected mainly in the outer membrane and the soluble protein fraction. We also found that the formation of protein aggregates and glycation products was inhibited by lower concentration (0.2%) of osmolytes: carnosine, glycine betaine and trehalose. Notably, supplementation of *E. coli* culture with higher osmolyte concentration (0.45% glycine betaine or trehalose) enhanced protein aggregation but reduced glycation and increased *E. coli* survival. Therefore, we concluded that glycation was the main cause of the loss of cell viability, whereas aggregates possibly played a protective function [19]. It is also worth mentioning that different osmolytes can stabilize or prevent the formation of liquid droplets, amorphous aggregates and amyloids. Opposite effects can be observed depending on the protein tested and osmolyte concentration [94].

For this review, we performed further analyses using different applications and algorithms, including PONDR (http://www.pondr.com, accessed on 20 May 2023), FuzDrop, (https://fuzdrop.bio.unipd.it/predictor, accessed on 10 May 2023 [95]), PSPredictor (http://www.pkumdl.cn:8000/PSPredictor, accessed on 10 May 2023 [96]) and the Disprot database, to better characterize the aggregates. To estimate the level of each protein in the aggregates, the emPAI values were used, and the total abundance of proteins was provided by the PaxDb database (*E. coli*-Whole Organisms, Integrated). Ribosomal proteins were the most abundant components of the aggregates, which contained a total of 547 proteins [19]. In addition to ribosomal proteins, the group of twenty main aggregate components included the elongation factor EF-Tu, tryptophanase TnaA and Dps (Table 2.).

The high level of these proteins in aggregates may reflect their high abundance in *E. coli* cells. However, the correlation between the total protein levels and their abundance in the aggregates (emPAI% values) was relatively moderate (Spearman’s coefficient of 0.47). As mentioned above, Dps may form coacervates with DNA or RNA in vitro [62]. In stationary *E. coli* cells, Dps coprecipitates with DNA [65] and can be isolated separately from other aggregated proteins using sucrose-density gradient centrifugation [97].

**Figure 2:**
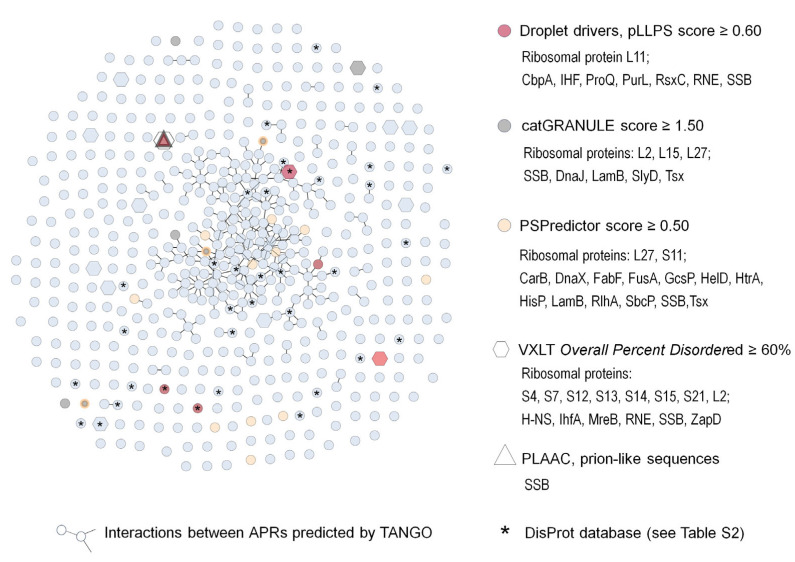
*E. coli* aggregates formed during desiccation–rehydration stress contain LLPS-prone proteins, IDPs and proteins interacting via APRs. The network was visualized with Cytoscape 3.9.1. program. Of the total 547 proteins, 216 interacted via APRs with at least one partner protein. APRs were identified by the TANGO algorithm (http://tango.crg.es/, accessed on 25 June 2023). To predict the propensity of the proteins to LLPS, the following algorithms were used: FuzDrop (https://fuzdrop.bio.unipd.it/predictor, pLLPS score, accessed on 10 May 2023), catGRANULE (http://s.tartaglialab.com, accessed on 15 May 2023) and PSPredictor (http://www.pkumdl.cn:8000/PSPredictor/, accessed on 10 May 2023). Intrinsically disordered and prion-like sequences were analyzed using PONDR (http://www.pondr.com/, VLXT predictor, accessed on 20 May 2023) and PLAAC applications (http://plaac.wi.mit.edu/, accessed on 10 May 2023), respectively. Proteins identified by each predictor are listed in the legend and marked with different shapes and colors.

Although previous studies indicated that the aggregates were enriched in proteins prone to LLPS [19], there was no correlation between the concentration of proteins in the aggregates and the tendency to LLPS predicted by catGranule, FuzDrop or PSPredictor (Spearman’s coefficient of −0.11, −0.04 and −0.06, respectively). Nevertheless, the algorithms identified multiple LLPS-prone proteins in the aggregates (Figure 2). The aggregates contained eight “Droplet drivers” according to the FuzDrop platform, 16 LLPS proteins detected by PSPredictor and eight LLPS-prone proteins with catGranule score ≥ 1.5. Only one protein, SSB, was identified by all three algorithms. SSB is also one of six *E. coli* prion-like proteins predicted by the PLAAC algorithm [98] and annotated in the DisProt database of experimentally confirmed IDPs. There are 121 *E. coli* IDPs in the DisProt database, of which 31 have been detected in the aggregates (Figure 2, Table S2.). The VLXT algorithm identified fourteen proteins with at least 60% disordered sequence. However, only four of them (L2, IHF, RNE, SSB) were classified as LLPS-prone proteins by at least one of the predictors. This result is not surprising because, apart from structural disorder, other parameters, including RNA-binding domains, amino acid patterns and IDRs position, determine the LLPS-tendency. Notably, most LLPS-prone proteins in the aggregates had at least 30% disordered sequence and at least one IDR longer than 35 amino acids. We also compared the composition of the aggregates and LLPS-dependent aggresomes described by Jin et al. [16,18]. Almost 60% of the aggresome proteins were detected in the aggregates. Altogether, these findings support our hypothesis that LLPS can promote the formation of protein aggregates during desiccation–rehydration stress.

We next identified APRs in aggregated proteins using the TANGO algorithm [70]. Similar to Khodoparast et al. [75], we selected six amino acid fragments with a TANGO score of at least 20%. Identical five-amino acid APRs and six-amino acid APRs with one mismatched residue were identified in different proteins and used to build the interaction network (Figure 2). We found that 216 proteins interacted via APRs with at least one partner. Further, 13 proteins, including GlpK, SucC, UvrB, WecA, DnaE, MurE, AcnB and GlnD, were connected with five or more (up to eight) other proteins. The other six proteins in this group were membrane proteins. We supposed that these proteins were trapped in the aggregates due to inner and outer membrane damage during desiccation. Ten proteins interacting via APRs were also classified as LLPS-prone proteins by at least one of the predictors.

In summary, these analyses suggest that protein aggregation in *E. coli* exposed to desiccation–rehydration stress may occur in a mixed manner, including LLPS and interactions between disordered sequences and APRs. We suppose that during the transition from the liquid to the solid (aggregated) state, separate condensates, oligomers or small aggregates can be recruited into the final aggregate (Figure 1C). It should be pointed out that the aggregates isolated after desiccation–rehydration may also be a mixture of various types of condensates formed in the cell: Dps-DNA complexes, liquid droplets and stable aggregates, each with potentially different protein composition. Apart from Dps-DNA, five other components of the aggregates: SSB, FtsZ and RNAP subunits (described in Section 3) may form separate condensates. Further experiments are needed to reveal the structure and complexity of these aggregates and to confirm the hypothesis that LLPS drives their formation during desiccation stress.

## 5. Concluding Remarks

Studying liquid phase separation of proteins and MLO formation in bacteria is a big challenge, mainly due to the small size of bacterial cells. However, developing high-resolution microscopy and single-molecule tracking techniques has enabled significant progress in this field in recent years. Information on LLPS-prone proteins and IDPs has been gathered in several rapidly growing databases, and over 100 predictors of disordered sequences have been developed in the last decade [99]. There are several examples of bacterial LLPS–dependent condensates that are involved in various physiological processes. There is also strong evidence that LLPS is an initial step in the formation of protein aggregates that enable bacteria to survive various stresses [16]. Different mechanisms of protection associated with aggregates (MLOs) are possible: (1) sequestration and protection of proteins against irreversible inactivation or degradation, (2) activation of the stress response including molecular chaperones, and (3) induction of a dormant state. It is worth noting that the bacterial cytoplasm has properties of glass-forming liquids and is denser than the intracellular environment in eukaryotic cells [100,101]. Moreover, inhibiting metabolic activity (e.g., during dormancy), may decrease the fluidity of the bacterial cytoplasm [101]. Therefore, the crowding effect which drives LLPS may be stronger in bacteria than in eukaryotic cells. On the other side, it may also accelerate the transition of condensates into nonfunctional amyloids or a solid-like state. Further studies on protein LLPS and condensation in bacteria are crucial for understanding mechanisms enabling the adaptation of bacteria to harmful conditions. It is particularly important in the case of clinical isolates and foodborne pathogens, which are able to survive in a desiccated state for extended periods.

## Figures and Tables

**Table 1:** Protective protein aggregates. See the text for more details.

Species	Conditions Inducing Aggregation	Protection Against	Proposed Mechanism of Protection	Comments	Ref.
*E. coli*	Sublethal heat stress, hydrogen peroxide, streptomycin	More severe heat shock	Induction of protein quality control components	“Memory” aggregates	[15]
*A. baumannii*	Desiccation, streptomycin, Δ*lon* mutation	Desiccation	Protection of sequestered proteins	Preserved activity of a model enzyme	[17]
*E. coli*	Stationary phase	Antibiotics	Dormancy		[84]
*E. coli* and other Gram-negative species	Stationary phase, heat shock, streptomycin, hydrogen peroxide	Antibiotics	Dormancy	MLOs	[16,18]
*E. coli*	Desiccation–rehydration	Desiccation–rehydration stress	Protection of sequestered proteins	Contain LLPS-prone proteins and IDPs	[19]

**Table 2:** The most abundant proteins detected in aggregates isolated from *E. coli* exposed to desiccation–rehydration stress. Supplementary Table S1 contains the complete list of proteins (≥0.2 emPAI %).

	ID	Protein Names	emPAI %	Abundance PaxDb (ppm)	catGranule	FuzDrop pLLPS	PSPredictor
1	P0AG51	50S ribosomal protein L30	2.88	6056	−0.970	0.117	0.005
2	P02413	50S ribosomal protein L15	2.82	3541	2.075	0.514	0.285
3	P62399	50S ribosomal protein L5	2.79	5965	0.283	0.114	0.002
4	P0CE47	Elongation factor Tu 1	2.25	27,871	0.850	0.147	0.043
5	P60422	50S ribosomal protein L2	1.60	1852	1.887	0.421	0.245
6	P61175	50S ribosomal protein L22	1.55	6098	−0.529	0.237	0.023
7	P02359	30S ribosomal protein S7	1.32	10,524	−0.038	0.310	0.004
8	P0AG55	50S ribosomal protein L6	1.25	3285	0.829	0.179	0.063
9	P0A7 × 3	30S ribosomal protein S9	1.23	1374	0.811	0.262	0.119
10	P0ADY3	50S ribosomal protein L14	1.19	4074	0.585	0.139	0.094
11	P0A7V0	30S ribosomal protein S2	1.18	3366	0.411	0.255	0.051
12	P0A7W7	30S ribosomal protein S8	1.09	2813	0.038	0.123	0.003
13	P0A7M2	50S ribosomal protein L28	1.00	4610	−0.283	0.142	0.013
14	P0A7S9	30S ribosomal protein S13	1.00	6249	0.322	0.245	0.014
15	P0A7V8	30S ribosomal protein S4	0.96	2549	0.690	0.178	0.003
16	P0A7R9	30S ribosomal protein S11	0.90	735	0.545	0.170	0.595
17	P68919	50S ribosomal protein L25	0.89	18,593	−0.224	0.144	0.006
18	P0A853	Tryptophanase, TnaA	0.89	830	0.538	0.141	0.005
19	P0ABT2	Dps	0.89	7698	−0.527	0.126	0.012
20	P60438	50S ribosomal protein L3	0.88	5500	1.392	0.235	0.103

## Data Availability

Not applicable.

## Supplementary Materials

The following supporting information can be downloaded at: https://www.mdpi.com/article/10.3390/molecules28186582/s1, Tables S1: Proteins aggregated in E. coli upon desiccation-rehydration stress; Table S2: E. coli IDPs annotated in the Disprot database and detected in the aggregates.
